# JDP2 and ATF3 deficiencies dampen maladaptive cardiac remodeling and preserve cardiac function

**DOI:** 10.1371/journal.pone.0213081

**Published:** 2019-02-28

**Authors:** Roy Kalfon, Tom Friedman, Shir Eliachar, Rona Shofti, Tali Haas, Lilach Koren, Jacob D. Moskovitz, Tsonwin Hai, Ami Aronheim

**Affiliations:** 1 Department of Cell Biology and Cancer Science, The B. Rappaport Faculty of Medicine, Technion-Israel Institute of Technology, Haifa, Israel; 2 Cardiac Surgery Department, Rambam Health Care Campus, Haifa, Israel; 3 The Pre-Clinical Research Authority Unit, The Technion, Israel Institute of Technology, Haifa, Israel; 4 Department of Biological Chemistry and Pharmacology, The Ohio State University, Columbus, Ohio United States of America; Scuola Superiore Sant'Anna, ITALY

## Abstract

c-Jun dimerization protein (JDP2) and Activating Transcription Factor 3 (ATF3) are closely related basic leucine zipper proteins. Transgenic mice with cardiac expression of either JDP2 or ATF3 showed maladaptive remodeling and cardiac dysfunction. Surprisingly, JDP2 knockout (KO) did not protect the heart following transverse aortic constriction (TAC). Instead, the JDP2 KO mice performed worse than their wild type (WT) counterparts. To test whether the maladaptive cardiac remodeling observed in the JDP2 KO mice is due to ATF3, ATF3 was removed in the context of JDP2 deficiency, referred as double KO mice (dKO). Mice were challenged by TAC, and followed by detailed physiological, pathological and molecular analyses. dKO mice displayed no apparent differences from WT mice under unstressed condition, except a moderate better performance in dKO male mice. Importantly, following TAC the dKO hearts showed low fibrosis levels, reduced inflammatory and hypertrophic gene expression and a significantly preserved cardiac function as compared with their WT counterparts in both genders. Consistent with these data, removing ATF3 resumed p38 activation in the JDP2 KO mice which correlates with the beneficial cardiac function.

Collectively, mice with JDP2 and ATF3 double deficiency had reduced maladaptive cardiac remodeling and lower hypertrophy following TAC. As such, the worsening of the cardiac outcome found in the JDP2 KO mice is due to the elevated ATF3 expression. Simultaneous suppression of both ATF3 and JDP2 activity is highly beneficial for cardiac function in health and disease.

## Introduction

The c-Jun dimerization protein (JDP2) is a member of the basic leucine zipper (bZIP) family of transcription factors [1,2], reviewed in [3]. JDP2 binds to the 12-O-tetradecanoylphorbol 13 acetate (TPA) response elements (TREs) and Cyclic AMP response elements (CREs) found in the regulatory region of numerous genes [1]. Upon binding, JDP2 typically represses transcription as a homodimer by recruitment of histone deacetylase proteins to the promoter region [4] and by competition with other transcription activators. Alternatively, JDP2 can dimerize with Chop10, another member of the bZIP family, and the resulting heterodimer activates transcription [5]. Functionally, JDP2 was found to play a role in cellular differentiation of skeletal muscle [6], adipocytes [7] and osteoclasts [8], as well as in other cellular processes including cell proliferation [9], nucleosome assembly [10] and cell senescence [11]. In addition, mice with inducible expression of JDP2 in their cardiomyocytes (expression driven by the αMHC promoter) had massive biatrial dilatation, atrioventricular conduction defect and increased mortality, suggesting that JDP2 is detrimental to the heart function [12,13]. If JDP2 is indeed detrimental, as suggested by the gain-of-function approach, one would expect the JDP2 KO mice to perform better than the WT mice under stress. Surprisingly, a loss-of-function approach showed an opposite adaptive role of JDP2. In a pressure overload model induced by Transverse Aortic Constriction (TAC), JDP2 KO mice performed worse than WT mice [14]. These results supported a protective role of JDP2, opposite from the expectation. In this report, we present data that not only provides a molecular explanation to this conundrum, but also shed light on the intricate interplay between JDP2 and its close homolog Activating Transcription Factor 3 (ATF3) reviewed in [15].

ATF3 shares 90% identity with JDP2 within the bZIP domain. Interestingly, JDP2 represses the expression of the ATF3 gene by binding to an ATF/CRE composite site within the ATF3 promoter [16]. Since JDP2 is expressed constitutively, its ability to repress the ATF3 gene would contribute to a low level of ATF3 expression under unstressed condition. Consistent with this idea, upon JDP2 loss-of-function, ATF3 basal expression is elevated [14,16]. Functionally, ATF3 has been shown to be detrimental to the heart in the context of chronic conditions. Mice with ATF3 expression in cardiomyocytes display maladaptive cardiac remodeling and reduced cardiac function [17,18]. Consistently, ATF3 deficiency provided partial protection under cardiac stress models in two pressure overload models: the phenylephrine infusion [19,20] and the TAC model [14]. Thus, ATF3 expression is detrimental to the heart function as evidenced by both the gain- and loss-of-function approaches. The maladaptive role of ATF3 in cardiac remodeling was challenged by other groups, suggesting a cardiac protective role of ATF3 [21–23]. These apparent discrepancies will be addressed in the Discussion.

In light of the elevated expression of ATF3 in the JDP2 KO mice and the detrimental consequences, we hypothesized that the deteriorated phenotype in the hearts of JDP2 KO mice is at least in part, mediated by ATF3. To test this hypothesis, we crossed JDP2 KO mice with ATF3 KO mice to generate JDP2/ATF3 double KO mice, designated hereafter: the dKO mice. As shown below, these dKO mice were resistant to maladaptive cardiac remodeling processes and exhibited preserved cardiac function, supporting the above hypothesis. The implication of these findings on the JDP2 and ATF3 interplay will be discussed.

## Materials and methods

### Mice

All animal studies have been approved by the Technion animal ethics committee and have therefore been performed in accordance with the ethical standards laid down in the 1964 Declaration of Helsinki and its later amendments. This study was carried out in strict accordance with the Guide for the Care and Use of Laboratory Animals of the National Institute of Health. In addition, our protocol was approved by the Committee of the Ethics of Animal Experiments of the Technion. All surgeries were performed under isoflurane anesthesia and all efforts were made to minimize mice suffering using Buprenorphine injection post-surgery (120 μg/Kg). The ATF3 gene is located on chromosome 1, whereas the JDP2 gene is located on chromosome 12. C57BL/6 mice with whole-body ATF3-KO [24] and JDP2-KO [7] were crossed in a ratio of female:male  =  2:1. This enabled the generation of double knock-out mice (designated hereafter dKO). The dKO mice were born in a Mendelian distribution, and display no overt phenotype. Male and female mice were used in all the experiments performed in this study and analyzed separately.

### TAC surgery

All experimental protocols were approved by the Institutional Committee for Animal Care and Use at the Technion, Israel Institute of Technology, Faculty of Medicine, Haifa, Israel. All study procedures were complied with the Animal Welfare Act of 1966 (P.L. 89–544), as amended by the Animal Welfare Act of 1970 (P.L.91-579) and 1976 (P.L. 94–279). Transverse aortic constriction (TAC) surgery was performed on male and female Wild type (WT) and dKO mice (10–12 weeks old) as described [14,25]. All TAC procedures along this study were performed by a single person blinded to the mice genotype.

### Magnetic resonance imaging (MRI) acquisition and analysis

Cardiac MRI was performed to measure cardiac function and determine the severity of the TAC surgery. Details of the MRI and all other related experimental methods were described previously [14]. EF was calculated as follows: EF (%) = [(LVEDV- LVESV)/ LVEDV] * 100.

### Echocardiography

Mice were anesthetized with 1% of isoflurane and kept on a 37°C heated plate throughout the procedure. An echocardiography was performed using a Vevo2100 micro-ultrasound imaging system (VisualSonics, Fujifilm) which was equipped with 13-38MHz (MS 400) and 22-55MHz (MS550D) linear array transducers. Those performing echocardiography and data analysis were blinded to the mice genotype. Cardiac size, shape, and function were analyzed by conventional two-dimensional imaging and M-Mode recordings. Maximal left ventricular end-diastolic (LVDd) and end-systolic (LVDs) dimensions were measured in short-axis M-mode images. Fractional shortening (FS) was calculated as follows: FS (%) = [(LVDd-LVDs)/LVDd] X 100. All values were based on the average of at least five measurements.

### Heart harvesting

Following eight weeks of TAC, mice were anesthetized, weighed and sacrificed. Hearts were excised, and ventricles were weighed and then divided into three pieces that were used for protein extraction, RNA purification, and histological analysis.

### mRNA extraction

mRNA was purified from ventricles using an Aurum total RNA fatty or fibrous tissue kit (#732–6830, Bio-Rad) according to the manufacturer’s instructions.

### Quantitative real time PCR (qRT-PCR)

cDNA was synthesized from 800 ng of purified mRNA derived from the ventricles. Purified mRNA was added to a total reaction mix of high-capacity cDNA reverse transcription kit (#4368814, Applied Biosystems) in a final volume of 20μl. Real-time PCR was performed using Rotor-Gene 6000TM (Corbett) equipment with absolute blue SYBR green ROX mix (Thermo Scientific AB-4162/B). Serial dilutions of a standard sample were included for each gene to generate a standard curve. Values were normalized to ubiquitin-conjugating enzyme E2D 2A (Ube2d2a) expression levels [26]. The primer sequences are shown in S1 Table.

### Cell size analysis

Heart tissue was fixed in 4% formaldehyde overnight, embedded in paraffin, serially sectioned at 10 μm intervals, and then mounted on slides. Sections were stained following deparaffinization with Wheat-germ agglutinin FITC-conjugated (Sigma Aldrich Cat# L4895) and diluted to a 1:100 phosphate-buffered saline (PBS). Sections were washed three times with PBS and mounted in Fluorescence Mounting Medium (Dako, S3023). Images were acquired by using panoramic flash series digital scanner (3DHistech Pannoramic 250 Flash III). Quantification of the cell size was performed with Image Pro Plus software. Five fields in each slide were photographed. Unstained areas were then identified and segmented using Image Pro Plus software. In each stained area, the mean cell perimeter and area was calculated, and the number of cells was measured.

### Fibrosis staining

Heart tissue was fixed in 4% formaldehyde overnight, embedded in paraffin, serially sectioned at 10 μm intervals, and then mounted on slides. Masson’s trichrome staining was performed according to the standard protocol. Images were acquired by using Virtual Microscopy (Olympus). The percent of the interstitial fibrosis was determined as the ratio of the fibrosis area to the total area of the heart section using Image Pro Plus software.

### Western blot analysis and quantification

Harvested tissues were homogenized in RIPA buffer (PBS containing 1% NP-40, 5 mg/ml Na-deoxycholate, 0.1% SDS) and supplemented with a protease inhibitor cocktail (P-8340, Sigma Aldrich). Homogenization was performed at 4°C using the Bullet Blender homogenizer (BBX24; Next advance) according to the manufacturer's instructions as previously described (Koren, 2015 #1364).

### Antibodies

The primary antibodies used: anti-phospho-ERK (Cat# M-9692) was purchased from Sigma Aldrich. Anti-p38 (Cat# 9212), anti-phospho-p38 (Cat# 9211) and anti-ERK (Cat# 9102) were purchased from Cell Signaling.

### Statistics

Our data is expressed as means ± SE. The comparison between several means was analyzed by one-way ANOVA followed by Tukey's post hoc analysis. All statistical analyses were performed using GraphPad Prism 5 software (La Jolla, CA). A P value ≤ 0.05 was accepted as statistically significant.

## Results

To test the hypothesis that elevated expression of ATF3 in JDP2-KO mice is responsible for the deteriorated cardiac phenotype following TAC, we deleted ATF3 in the JDP2-KO background by crossing the JDP2 KO with the ATF3-KO mice to generate the whole body dKO mice.

### Analysis of cardiac hypertrophy at basal

**Fig 1:**
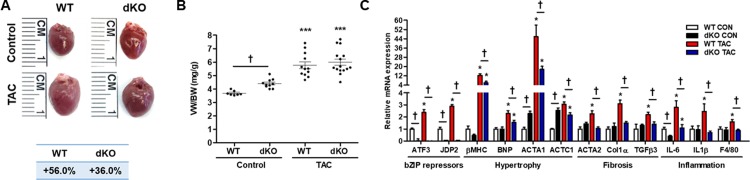
dKO male mice display attenuated cardiac hypertrophy following TAC. Male mice were treated with TAC for 8 weeks and their hearts were analyzed. **A** Representative pictures of control and TAC-operated mice hearts of each genotype. The percentage increase in ventricles weight (VW) to mouse body weight (BW) ratio (mg/gr) by TAC is shown at the bottom. **B** The ratio of VW/BW is shown (n = 7-15/group). **C** mRNA was extracted from ventricles and the expression level of cardiac remodeling, hypertrophic and inflammatory markers were measured by qRT-PCR. Expression levels are presented as relative values (compared to wild type control mice, defined as 1, n = 6-8/group). All results represent the mean ± SE ^***^P ≤ 0.05, control vs. TAC; ^†^*P* ≤ 0.05, difference between genotypes.

We first examined the mice under control (unstressed) condition. Hearts from 20-weeks-old dKO male mice were bigger in size and had a slightly higher (statistically significant, *P<*0.05) ventricular weight/body weight (VW/BW) ratio than the WT male mice (Fig 1). While the VW of both mice genotypes was not different, the basal BW of dKO mice strain was significantly lower (S1 Fig). Indeed, no significant increase was observed in hypertrophic markers associated with maladaptive remodeling, such as βMHC or BNP (Fig 1C). We next examined whether the higher VW/BW found in dKO male mice is gender-specific by examining the female mice. As shown in S2 Fig, female mice showed no difference in basal VW/BW ratio as well as BW and VW between WT and dKO mice. Thus, only male mice had a slight increase VW/BW ratio, which corresponded mainly to the lower BW. Consistently, the expression levels of two sarcomeric actin isoforms, ACTA1 and ACTC1, were significantly elevated in dKO male mice as compared with their WT counterparts (Fig 1C), whereas in female hearts the hypertrophic and sarcomeric markers were similar between WT and dKO (S2 Fig).

### Analysis of cardiac hypertrophy following TAC

To test the role of dual deficiency in JDP2 and ATF3 expression in stress-induced cardiac remodeling, we exposed 12-week-old mice to TAC for 8 weeks before analyses. To reveal the potential role of ATF3 and JDP2, we first examined their expression levels following TAC by qRT-PCR (Fig 1C). As previously shown, both JDP2 and ATF3 expression levels were elevated, whereas, in dKO mice no expression was observed. In males, hearts size and VW/BW ratio was significantly increased in both WT and dKO mice (Fig 1A and 1B). However, due to the higher basal VW/BW ratio in dKO mice, the calculated percentage increase was higher in WT than dKO mice: 56% versus 36%. (Fig 1A and 1B). In female mice, TAC resulted in increased heart size and VW/BW ratio in both genotypes and again with a statistical significant higher impact on the WT than dKO mice: 93% versus 52% (S2 Fig). The increase in heart size following TAC was accompanied by an elevation of hypertrophic markers, such as, βMHC, BNP, ACTA1 and ACTC1 in both genotypes (Figs 1C and S2). Consistent with the reduced severe phenotype in dKO, the increase in hypertrophic markers of TAC-operated dKO mice was significantly lower as compared with the WT counterparts in both genders (Figs 1C and S2). Interestingly, while in WT male mice the expression of the ACTC1, the abundant cardiac actin isoform, was highly elevated following TAC, no increase in ACTC1 expression was observed in dKO male mice (Fig 1C). Suggesting that no further increase was necessary in this sarcomeric protein to cope with the pressure overload condition in the dKO mice hearts.

**Fig 2:**
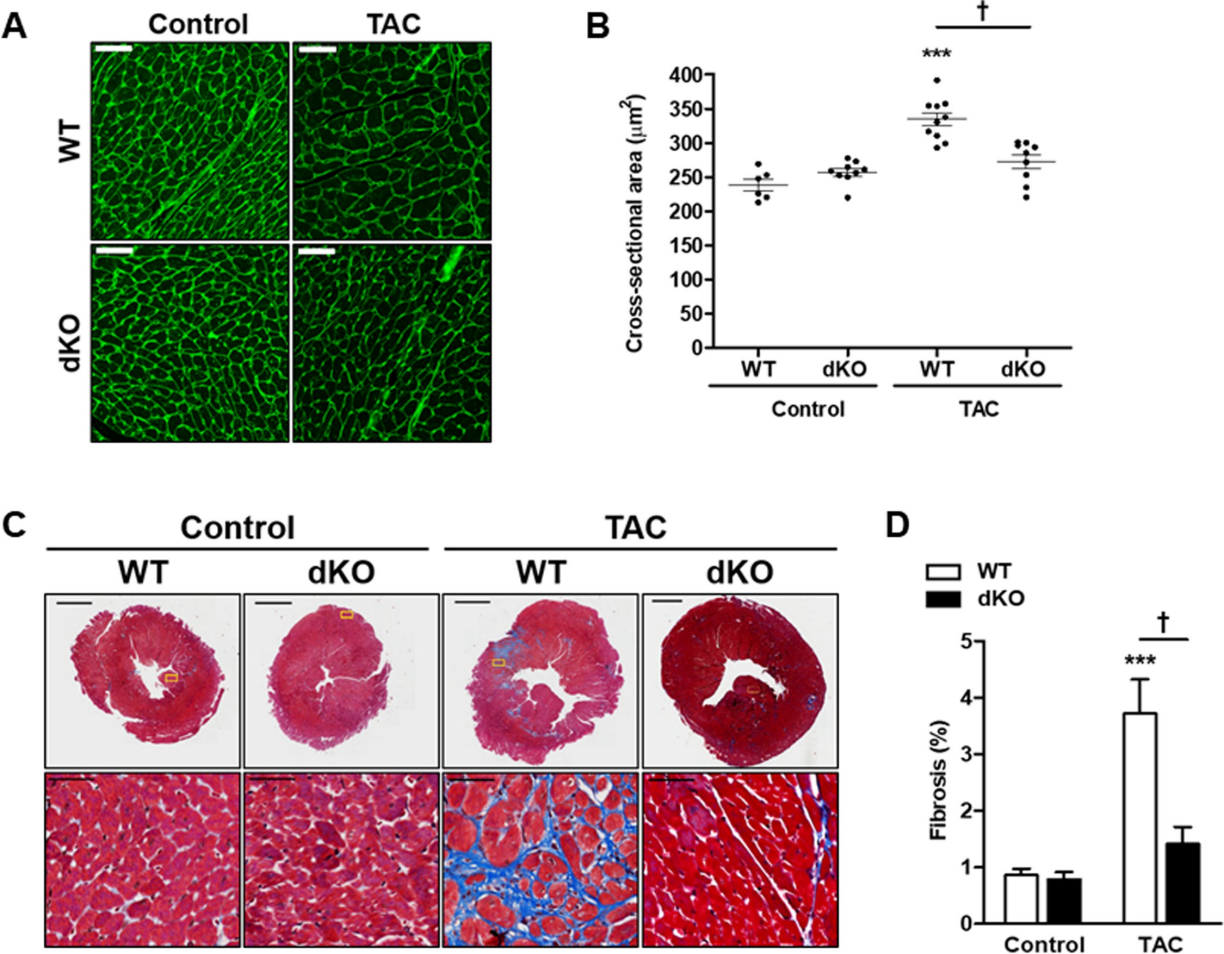
dKO male mice display attenuated cardiac fibrosis following TAC. Male mice were treated with TAC for 8 weeks and their hearts were analyzed. **A** Ventricles sections were stained with FITC-labeled wheat germ agglutinin and cell size was analyzed. Scale bar = 100 μm. **B** Quantification of cell size from D represented as cross sectional area in μm^2^. **C** Representative paraffin-embedded heart sections stained with Masson’s trichrome to visualize fibrosis. **D** Quantification of the level of fibrosis (%) stained by Masson's trichrome (n = 6-8/group). All results represent the mean ± SE ^***^P ≤ 0.05, control vs. TAC; ^†^*P* ≤ 0.05, difference between genotypes.

To assess the size of cardiomyocytes following TAC, we stained heart sections by fluorescently labeled wheat germ agglutinin to delineate the cell boundary, and calculated cardiomyocyte cross sectional area (CSA) of control and TAC-operated mice. In both genders, WT mice showed an increase of cardiomyocyte CSA by about 50% following TAC, but the dKO mice showed no significant increase (Figs 2A and 2B and S2).

### Analyses of fibrosis and inflammatory markers

We next examined cardiac fibrosis as part of cardiac remodeling hallmark. Quantitative analysis of fibrosis showed no difference between the genotypes at baseline (Figs 2C and 2D and S2). However, hearts derived from TAC-operated WT mice displayed a 4-fold increase, while dKO mice had only a mild increase (not statistically significant) in cardiac fibrosis (Fig 2C and 2D). Similar results were observed in female mice (S2 Fig). The increase in fibrosis in TAC-operated WT mice was accompanied by significantly elevated transcripts of fibrosis genes in both genders such as, ACTA2, ColIα and TGFβ3. Consistently, the transcripts of these markers did not increase in TAC-operated dKO mice in both gender (Figs 1C and S2).

We next examined the inflammatory response of the heart following TAC by examining IL-6 and IL-1β inflammatory markers, and F4/80, the marker for macrophages. All three markers were lower in TAC-operated dKO male mice than in the WT counterparts (Fig 1C). The dampened inflammatory response is consistent with the milder hypertrophy and fibrosis observed in dKO mice. Similarly, IL-6 transcription was not elevated in female dKO mice (S2 Fig).

**Fig 3:**
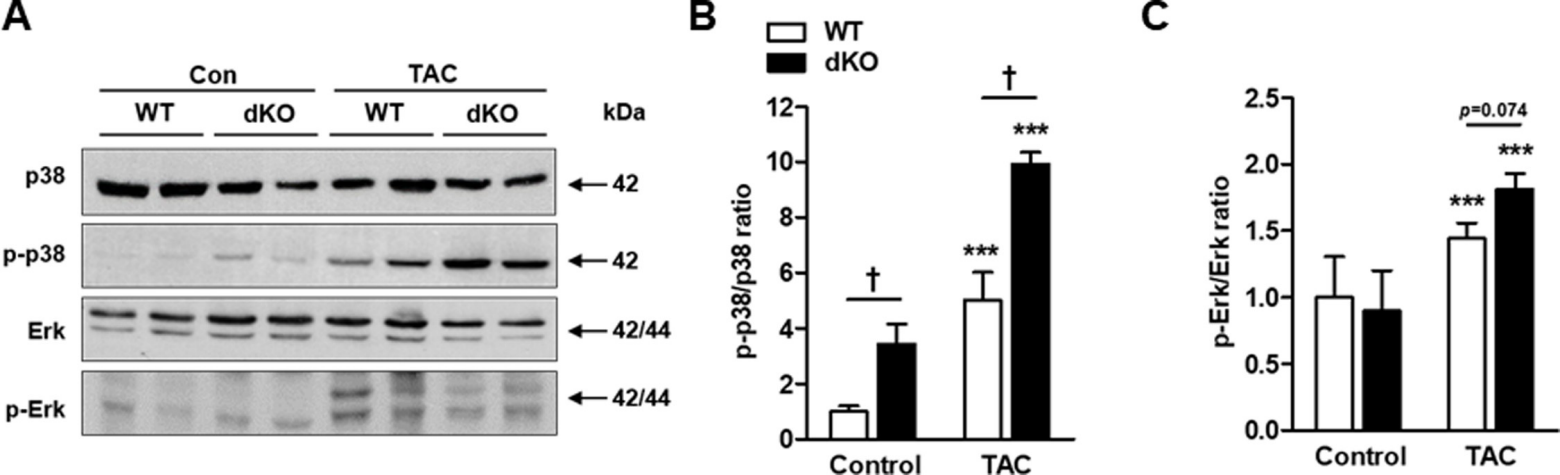
dKO male mice display increased p38 activity. Cardiac hypertrophy was induced by TAC in male mice. Eight weeks following TAC, mice were sacrificed and hearts were excised **A** Western blot analysis of heart lysate derived from the indicated genotypes with the indicated antibodies. **B-C** Densitometric analysis of Western blot shown in A is presented as mean ratio of the corresponding phospho-protein to total protein ± SE (compared to wild type control, defined as 1, n = 5-6/group). **B** pp38/p38. **C** pERK/ERK. ^***^P ≤ 0.05, control vs. TAC; ^†^*P* ≤ 0.05 difference between genotypes.

In our previous analyses of JDP2 KO mice, the activation of p38 was completely lost following TAC, and this lack of p38 activation correlated with maladaptive cardiac remodeling in these mice [14]. Thus, we examined the activation state of p38 by immunoblot. At baseline, we observed a higher phospho-p38/p38 ratio in the hearts of dKO mice as compared with WT (Fig 3A and 3B). Following TAC, p38 activation increased in both groups, but was more pronounced in the dKO mice (Fig 3A and 3B, a 10-fold versus 5-fold increase). Thus, following TAC, the lack of p38 activation, a feature that correlated with maladaptive cardiac remodeling in the hearts of JDP2 KO mice [14], was fully eliminated in the dKO mice (Fig 3A and 3B). Interestingly, TAC activated the extracellular regulated kinase (ERK) independent of the WT versus dKO genotype (Fig 3A and 3C), a result similar to our previous data that was independent of single deletion of either ATF3 or JDP2. Thus, these two bZIP genes had no impact on ERK activation by TAC.

### Analyses of cardiac function: The JDP2/ATF3 dKO mice performed better than the WT mice under TAC

**Fig 4:**
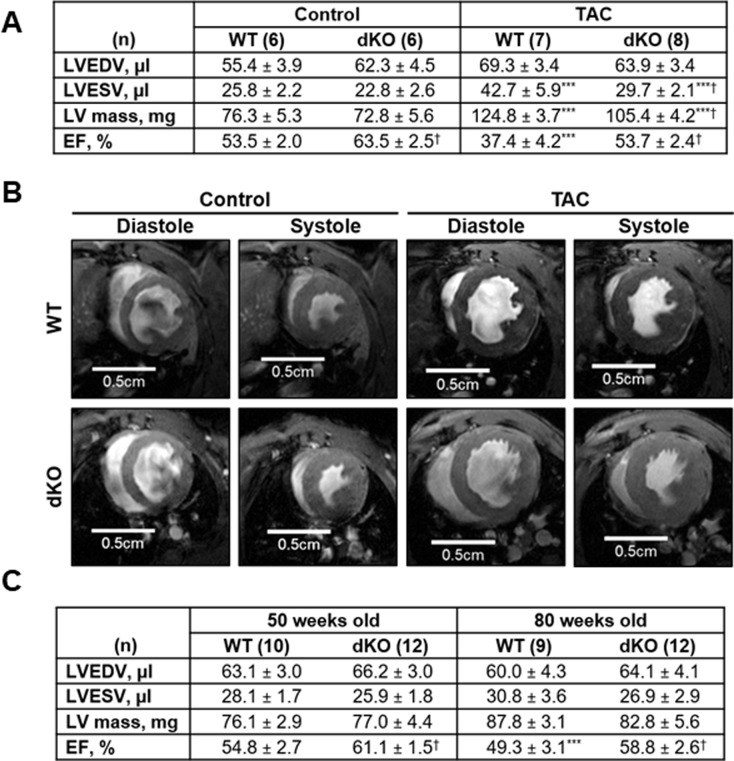
dKO male mice preserve contractile function following TAC. Cardiac hypertrophy was induced by TAC in male mice. Eight weeks following TAC, left ventricular cardiac volumes, mass and function were examined by a cardiac MRI. **A** The following parameters were measured: left ventricular (LV) mass, left ventricular end-diastolic (LVEDV) and left ventricular end-systolic volume (LVESV), and ejection fraction (EF) was calculated. The results represent the mean ± SE of the indicated number (n) of animals per group. ^***^P ≤ 0.05, control vs. TAC; ^†^*P* ≤ 0.05, difference between genotypes. **B** Representative images of mid-ventricular short-axis slice at peak diastole and systole. **C** Age-related decline in cardiac function was assessed at 50- and 80-weeks-old mice. Results were compared with control mice (20 weeks old). Left ventricular cardiac volumes, mass and function were examined by a cardiac MRI as described in A. The results represent the mean ± SE of the indicated number (n) of animals per group. ^***^P ≤ 0.05, control vs. TAC; ^†^*P* ≤ 0.05, difference between genotypes. ^***^P ≤ 0.05, different from 20- and 50-weeks-old mice; ^†^*P* ≤ 0.05, difference between genotypes.

Maladaptive cardiac remodeling characterized by hypertrophy, inflammation and fibrosis is associated with reduced cardiac function. To assess cardiac contractile function, we used MRI to calculate ejection fraction (EF) in control and TAC-operated male mice. The calculated EF in control mice suggests an improved basal contractile function in the dKO mice (higher EF than WT) at 20 weeks of age (Fig 4A and 4B). To assess the long-term effect of JDP2 and ATF3 deficiency on cardiac function, we measured the EF of 50 and 80 weeks old mice. An improvement of 10–20% in the calculated EF in dKO mice was preserved for at least 80 weeks (Fig 4C). We next tested cardiac volumes, function and mass following TAC. Indeed, TAC induced cardiac morphological changes (as shown by ventricular dilation and increased mass) and led to reduced cardiac function (as shown by reduced EF). However, these changes were quite different between genotypes. Consistent with the greater increase in VW/BW ratio by TAC in the WT male mice, the increase in left ventricular (LV) mass by TAC was significantly higher in the WT mice than that in the dKO mice: 64% versus 45% (Fig 4A). The hearts derived from TAC-operated WT mice showed a dilated phenotype with LV end diastolic volume (LVEDV) of 69.3 μl after TAC as compared with 55.4 μl at baseline (Fig 4A). In contrast, LVEDV of TAC-operated dKO mice were 63.9 μl, which was very similar to that at baseline: 62.3 μl (Fig 4A). In addition, the LV end systolic volume (LVESV) was significantly increased by TAC in both genotypes; however, the increase was significantly higher in the WT mice than dKO mice (65% versus 30%), indicating that the WT heart was less effective during systole (Fig 4A). As expected, EF was highly reduced in WT TAC-operated mice as compared to their control counterparts (-30%). Interestingly, TAC-operated dKO mice exhibited only a modest reduction in EF (-15%). In fact, the absolute EF value following TAC of dKO mice was similar to the EF obtained in control (unstressed) WT mice (Fig 4A). We also examined cardiac function in the female mice by echocardiography and calculated the fractional shortening (FS). At basal, no significant differences in FS was observed between WT and dKO control mice (S3 Fig). Consistent with the male mice findings, we found that in WT TAC-operated females the FS was highly reduced. It declined from ~28% to 15%, while in the dKO mice the FS was preserved (from ~30% to 26%, a reduction with no statistically significance) and indistinguishable from control WT mice (S3 Fig).

Collectively, following TAC, the hearts derived from both WT and dKO mice underwent hypertrophy, yet, the hearts derived from dKO mice showed reduced cardiac hypertrophy and suppressed maladaptive remodeling processes with highly preserved contractile function as compared with WT mice in both genders.

## Discussion

JDP2 and ATF3 are bZIP transcription factors that share 90% homology in their bZIP region. Both proteins can form heterodimers with other bZIP family members and can either suppress or activate transcription as homodimers or heterodimers in a context-dependent manner. A key difference between them is their bioavailability and mode of regulation. Whereas JDP2 is ubiquitously expressed, ATF3 is an immediate-early gene that is normally expressed at a low or undetectable level, but is highly induced by numerous stress signals. Interestingly, these proteins regulate the expression of each other [14,16]. Therefore, deficiency in either one of them results in an elevated expression of the other gene. Thus far, each gene has been shown to play a role in a variety of pathophysiological contexts using various mouse disease models such as cancer, neurodegeneration, diabetes, atherosclerosis, and heart failure (for reviews, see [3,15]). Among these, cardiac disease is a model that we have been using to investigate JDP2 and ATF3. Using a gain-of-function approach, we showed that transgenic mice ectopically expressing either JDP2 or ATF3 displayed maladaptive cardiac remodeling and hypertrophy [12,17,18]. The effects were independent of developmental events, since hypertrophic cardiac growth was observed following expression in adult mice using an inducible tet-off system [18,27]. We further investigated their roles in the heart using a loss-of-function approach.

**Fig 5:**
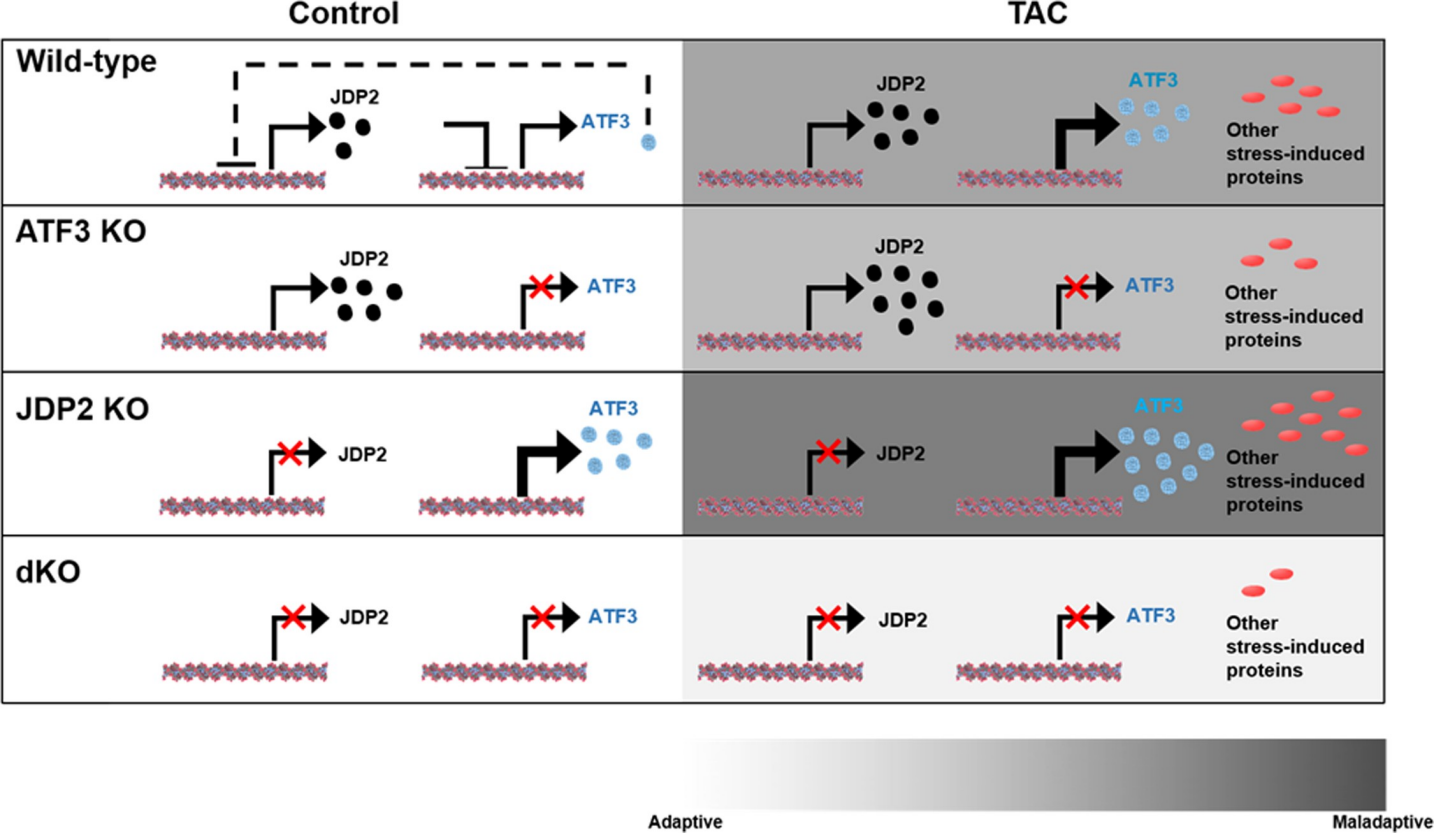
The dual loss of ATF3 and JDP2 model in cardiac remodeling. Schematic diagram showing the interplay between JDP2 and ATF3 in various mouse strains used in this and previous manuscript and the cardiac consequences in maintaining heart function in health (left panels) and following TAC (right panels). JDP2 and ATF3 protein expression levels are represented by black and light-blue circles, respectively. Other stress induced proteins are shown in red ovals. The panels represent the following mice strains: WT, ATF3 KO, JDP2 KO and dKO. Color code scale bar representing cardiac remodeling from adaptive to maladaptive is shown at the bottom (white to dark-grey respectively).

Consistent with the detrimental role of ATF3, its deletion afforded partial cardiac protection in the ATF3 KO mice in phenylephrine infusion model [19,20], while in the TAC model, ATF3 had a very mild beneficial outcome compared with WT mice [14]. In contrast, JDP2 deletion resulted in deterioration of cardiac function following TACA possible explanation for this discrepancy is that JDP2 overexpression mimics ATF3 function due to their high sequence homology. On the other hand, JDP2 deficiency results in elevated expression of ATF3, which was previously shown to promote cardiac maladaptive remodeling as well [18]. Therefore, both JDP2 overexpression and deficiency results in a net elevation of bZIP repressor activity. This may alter the delicate equilibrium between numerous bZIP family members resulting in a deteriorated outcome. Indeed, in the present study we demonstrate that JDP2 KO mice lacking ATF3 display improved cardiac outcome with preserved contractile function, supporting the above hypothesis. These results were observed in both male and female dKO mice and were significantly different than the expected additive mixed single KO genotypes. The interplay between JDP2 and ATF3 single KOs and dKO and their role in cardiac adaptation or maladaptation under stress is summarized (Fig 5). dKO mice display a better outcome in all molecular and physiological parameters used to assess cardiac remodeling and hypertrophy. This include hypertrophic markers, fibrosis, immune response, and cardiac function. Importantly, when we compare the calculated EF for all four genotypes namely; ATF3 KO and JDP2 KO mice from our previous article [14] in comparison to WT and dKO mice following TAC, the EF of dKO mice is significantly better than the EF of the single KOs of both ATF3 KO and JDP2 KO mice and is similar to the EF representing un-operated WT mice.

Since the dKO mice are deficient of JDP2 and ATF3 upon fertilization, one caveat is that the improved cardiac performance is due to some yet unidentified developmental beneficial effects, rather than better adaptation to the TAC stress. To address this issue, we analyzed the mice under un-stressed condition. In dKO male mice displayed higher VW/BW ratio than the WT mice. The higher VW/BW ratio in males is due to lower BW and is not observed in female mice. Functionally, dKO mice showed improved cardiomyocyte contractile function when compared with WT mice in both gender. This improvement was sustained in older mice at 50 and 80 weeks of age as well. In contrast, in the females VW/BW ratio, cardiac function and sarcomeric actin levels were indistinguishable between the genotypes; yet, following TAC, the dKO females displayed a cardiac protective phenotype. Thus, the beneficial phenotype that we observed following TAC in the dKO mice is independent of their basal cardiac function, making it unlikely to exhibit cardiac protection due to some unspecified developmental benefits.

We note that, in an apparent contradiction, two studies showed that ATF3 deficiency resulted in a deteriorated phenotype under TAC [21–23]. As we discussed previously [28], this discrepancy was thought to be due to the timing of analyses after TAC. We examined the mice at 8 weeks post TAC [14], while the others at 4 weeks [21,22]. It is well known that cardiac stress initially induces an adaptive response aiming to preserve cardiac function; however, when stress becomes chronic, the adaptive process turns into a maladaptive one. This fits well with our current understanding of the ATF3 biology. ATF3 is a stress gene induced by a long list of signals that disturb cellular homeostasis [29]. On the one hand, its induction under acute conditions appears to be beneficial, facilitating the cells to adapt. On the other hand, its expression under chronic conditions almost invariably leads to pathological consequences. As an example, acute induction of ATF3 in the pancreatic beta cells upon exposure to glucose increases their ability to up-regulate insulin gene expression and subsequent secretion. However, chronic induction of ATF3 leads to beta cell apoptosis [30]. Thus, the potential dichotomous role of ATF3 under acute versus chronic stress may be an explanation for the apparent discrepancy in the literature (above).

Both JDP2 and ATF3 are transcription factors. Clearly, an important mechanistic question is “what are the functionally relevant downstream targets for ATF3 and JDP2 in the context of cardiac stress?” It appears that the activity of the p38 signaling pathway plays a significant role and positively correlates with the cardiac function. Previously, we showed that the p38 pathway was completely abrogated in JDP2 deficient mice following TAC [14]. However, the present study showed a resumption of the p38 activation in the dKO mice. In addition, the level of p38 activation in the dKO mice was higher than that in the WT mice with or without TAC, and is correlated with the beneficial cardiac outcomes [31].

Although much advance is made through the use of genetically modified mice, compensatory mechanisms can obscure interpretation and may not truly represent the functional role of the targeted molecule. The identification of such compensatory mechanisms in the future is crucial for better understanding the complex interplay between key regulatory molecules.

In summary, we suggest that JDP2 and ATF3 double deficiency correlates positively with p38 activation and afforded a beneficial cardiac effect in both genders in response to pressure overload. Current treatments for heart failure are very limited. The inhibition of both JDP2 and ATF3, or the activation of p38 in the heart may serve as promising means to reduce maladaptive cardiac remodeling and improve cardiac function.

## Supporting information

S1 Fig dKO male mice display attenuated cardiac hypertrophy following TAC. Male mice were treated with TAC for 8 weeks and their hearts were analyzed. **A** Mice body weight (BW). **B** Mice ventricles weight (VW). All results represent the mean ± SE. ^***^P ≤ 0.05, control vs. TAC; ^†^*P* ≤ 0.05, difference between genotypes.
(TIF)

S2 Fig dKO female mice display reduced cardiac hypertrophy and fibrosis following TAC. Cardiac hypertrophy was induced by TAC in female mice. Eight weeks following TAC, mice were sacrificed and hearts were excised. **A** The ratio (mg/gr) of ventricles weigh (VW) to mouse body weight (BW) VW/BW (mg/gr) is shown. **B** Mice BW. **C** Mice VW. **D** mRNA was extracted from ventricles and the expression level of cardiac remodeling and hypertrophic, fibrosis and inflammatory markers were measured by qRT-PCR. Expression levels are presented as relative values (compared to wild type control mice, defined as 1, n = 6-8/group). **E** Ventricles sections were stained with FITC-labeled wheat germ agglutinin and the quantification of cross sectional area in µm^2^ is shown **F** Paraffin-embedded heart sections stained with Masson’s trichrome to visualize fibrosis and the level of fibrosis (%) was quantified (n = 6-8/group). All results represent the mean ± SE ^***^P ≤ 0.05, control vs. TAC; ^†^*P* ≤ 0.05, difference between genotypes.
(TIF)

S3 Fig dKO female mice preserve contractile function following TAC. Cardiac hypertrophy was induced by TAC in female mice. Eight weeks following TAC, mice hearts were examined by micro ultrasound. The following parameters were measured: interventricular septal end diastole (IVSd); left ventricular posterior wall end diastole (LVPWd); maximal left ventricular internal end-diastole (LVIDd); end-systole (LVIDs); and fractional shortening (FS). FS was assessed according to: FS (%) = [(LVDd-LVDs)/LVDd] * 100. All results represent the means ± SE of the indicated number (n) of animals per group. ^***^P ≤ 0.05, control vs. TAC; ^†^*P* ≤ 0.05, difference between genotypes.
(TIF)

S1 Table Oligonucleotide primers used for qRT-PCR analysis. (TIF)
