# Supplementary figures and images for: JDP2 and ATF3 deficiencies dampen maladaptive cardiac remodeling and preserve cardiac function
Source: PLoS One. 2019 Feb 28;14(2):e0213081. doi: 10.1371/journal.pone.0213081 (PMC6394944; doi:10.1371/journal.pone.0213081)

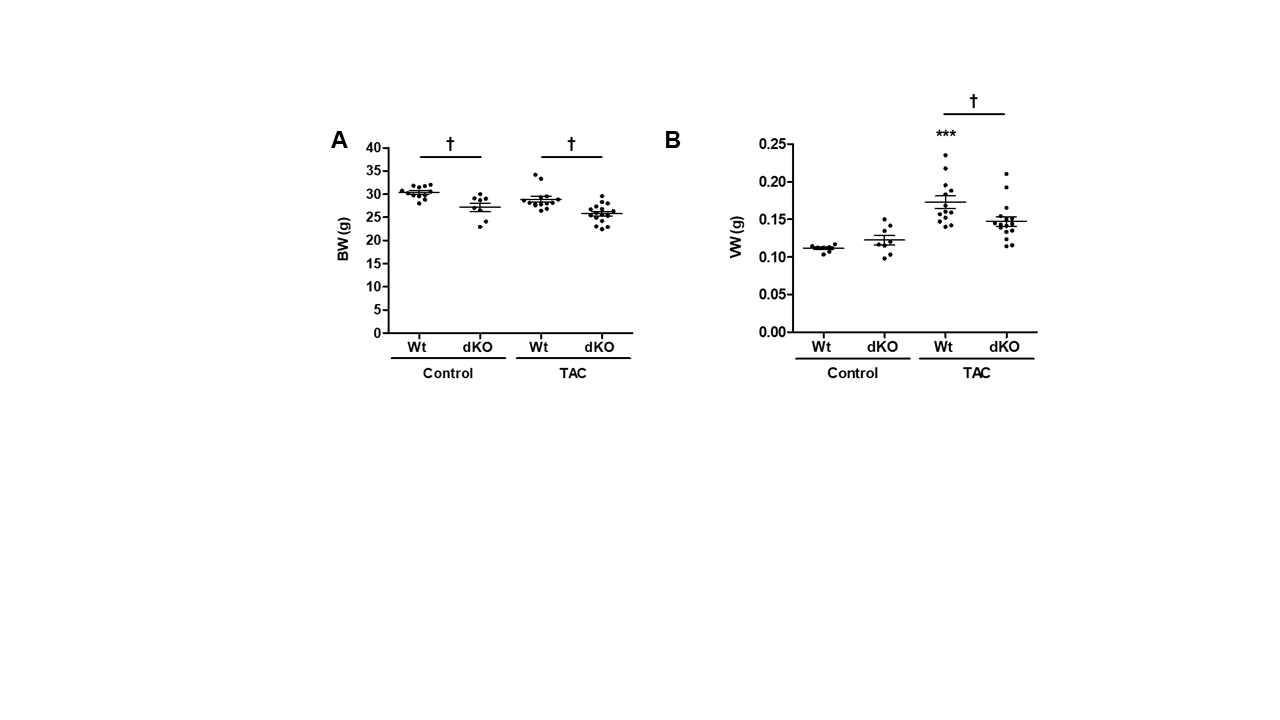

Supplement: S1 Fig — Male mice were treated with TAC for 8 weeks and their hearts were analyzed. A Mice body weight (BW). B Mice ventricles weight (VW). All results represent the mean ± SE. ***P ≤ 0.05, control vs. TAC; †P ≤ 0.05, difference between genotypes. (TIF) [file pone.0213081.s001.TIF]

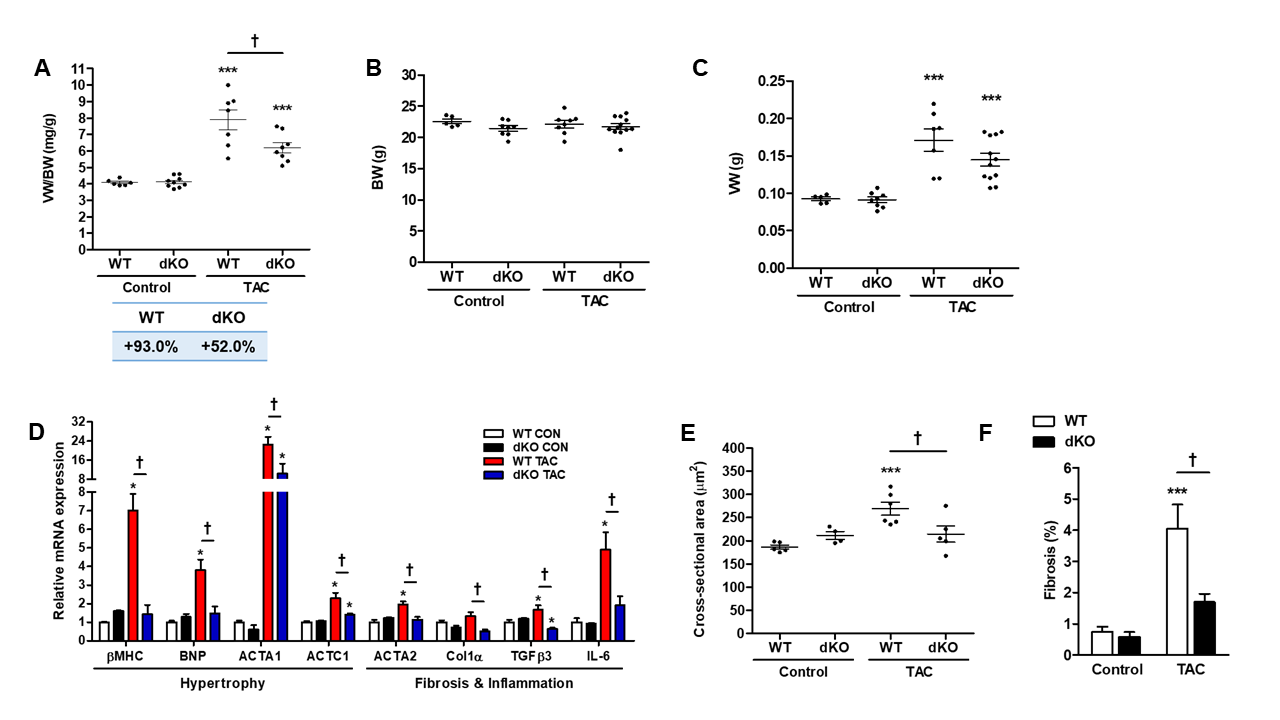

Supplement: S2 Fig — Cardiac hypertrophy was induced by TAC in female mice. Eight weeks following TAC, mice were sacrificed and hearts were excised. A The ratio (mg/gr) of ventricles weigh (VW) to mouse body weight (BW) VW/BW (mg/gr) is shown. B Mice BW. C Mice VW. D mRNA was extracted from ventricles and the expression level of cardiac remodeling and hypertrophic, fibrosis and inflammatory markers were measured by qRT-PCR. Expression levels are presented as relative values (compared to wild type control mice, defined as 1, n = 6-8/group). E Ventricles sections were stained with FITC-labeled wheat germ agglutinin and the quantification of cross sectional area in µm2 is shown F Paraffin-embedded heart sections stained with Masson’s trichrome to visualize fibrosis and the level of fibrosis (%) was quantified (n = 6-8/group). All results represent the mean ± SE ***P ≤ 0.05, control vs. TAC; †P ≤ 0.05, difference between genotypes. (TIF) [file pone.0213081.s002.tif]

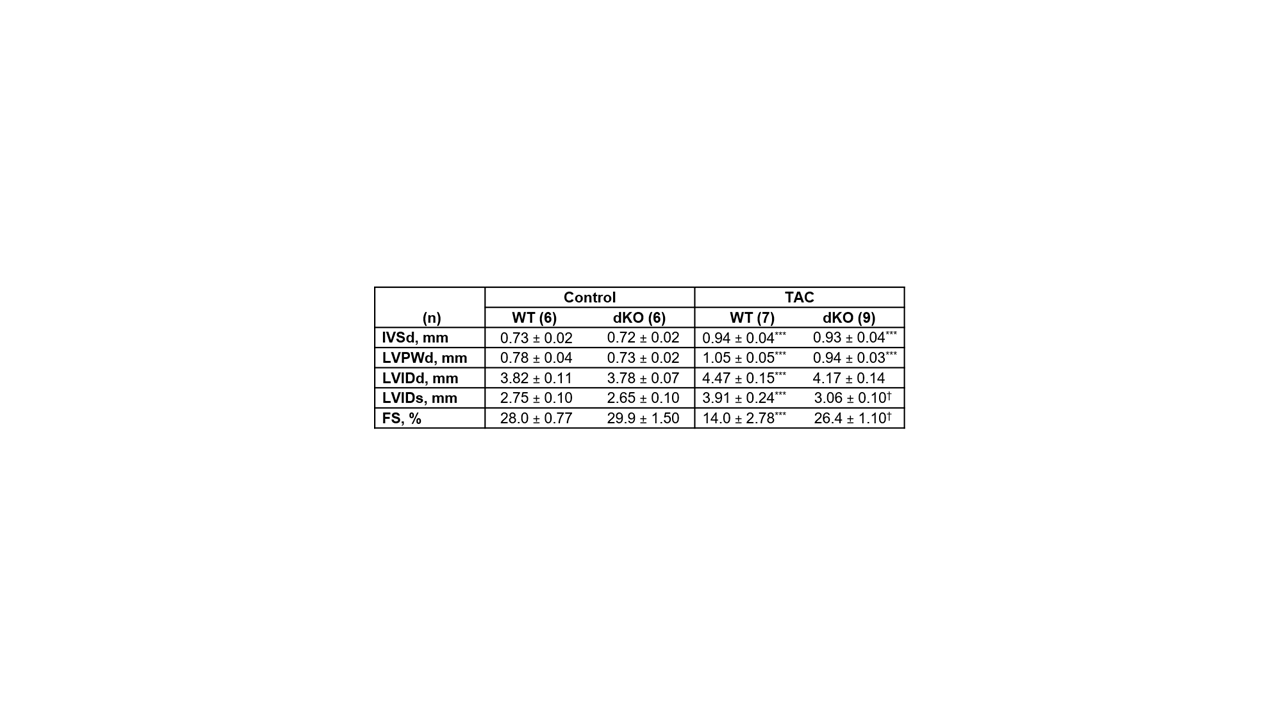

Supplement: S3 Fig — Cardiac hypertrophy was induced by TAC in female mice. Eight weeks following TAC, mice hearts were examined by micro ultrasound. The following parameters were measured: interventricular septal end diastole (IVSd); left ventricular posterior wall end diastole (LVPWd); maximal left ventricular internal end-diastole (LVIDd); end-systole (LVIDs); and fractional shortening (FS). FS was assessed according to: FS (%) = [(LVDd-LVDs)/LVDd] * 100. All results represent the means ± SE of the indicated number (n) of animals per group. ***P ≤ 0.05, control vs. TAC; †P ≤ 0.05, difference between genotypes. (TIF) [file pone.0213081.s003.tif]

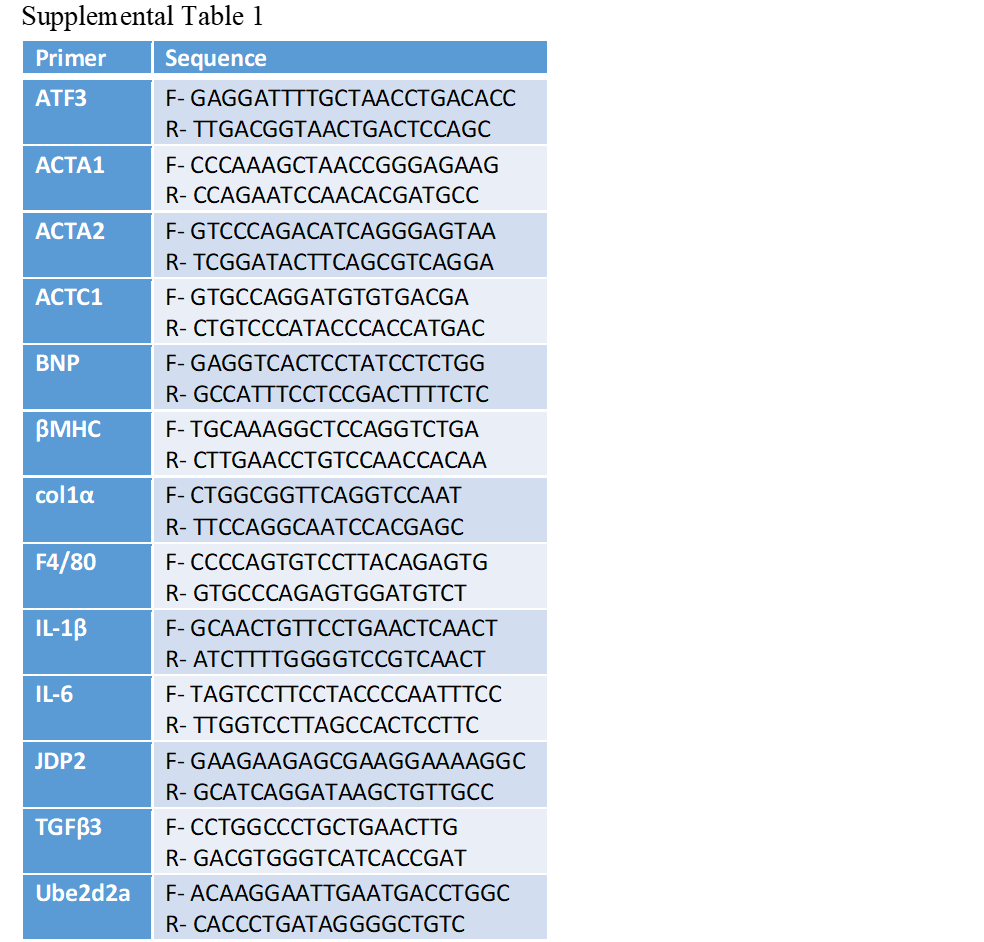

Supplement: S1 Table — (TIF) [file pone.0213081.s004.tif]
